# Quantifying the Relationship Between Financial News and the Stock Market

**DOI:** 10.1038/srep03578

**Published:** 2013-12-20

**Authors:** Merve Alanyali, Helen Susannah Moat, Tobias Preis

**Affiliations:** 1 Centre for Complexity Science, University of Warwick, Coventry, CV4 7AL, UK; 2 Warwick Business School, University of Warwick, Coventry, CV4 7AL, UK

## Abstract

The complex behavior of financial markets emerges from decisions made by many traders. Here, we exploit a large corpus of daily print issues of the *Financial Times* from 2^nd^ January 2007 until 31^st^ December 2012 to quantify the relationship between decisions taken in financial markets and developments in financial news. We find a positive correlation between the daily number of mentions of a company in the *Financial Times* and the daily transaction volume of a company's stock both on the day before the news is released, and on the same day as the news is released. Our results provide quantitative support for the suggestion that movements in financial markets and movements in financial news are intrinsically interlinked.

The movements of stock markets impact the lives of many individuals, within the financial sector and far beyond. Obvious benefits therefore lie in an improved understanding of the behavior of this complex system. Research towards this goal has been fuelled by the vast amount of data on financial transactions recorded at exchanges, with increasing numbers of studies in complex systems science aiming to analyze^1^,^2^,^3^,^4^,^5^,^6^,^7^ and model stock market behavior^8^,^9^,^10^,^11^,^12^.

Financial transaction data sets reflect the final outcome of a trader's decision making process^13^, the decision to buy or sell a particular stock. Such decisions may be influenced by various types of information in a trader's environment. In modern society, our interactions with the Internet are generating large new data sources on our consumption of information^14^,^15^,^16^,^17^,^18^. Previous work has demonstrated that search patterns on *Google* can be linked to various indicators of behavior in the real world^19^, such as reports of infections of influenza-like illnesses^20^, the economic success of nations^21^, and various other economic indicators such as popularity of international travel destinations and unemployment claims^22^.

Recent research has sought to investigate whether data on what information users seek online can provide insight into market movements. Preis, Reith and Stanley provided initial evidence of a link between online searches and financial market behavior, describing a correlation between the weekly number of *Google* searches on a company name and weekly cumulative transaction volume of the corresponding company's stock^23^. Preis, Moat and Stanley built on this result, demonstrating that changes in *Google* query volume for search terms related to finance could be interpreted as early warning signs of stock market moves^24^. Moat et al. showed that data on views of *Wikipedia* pages can also be related to market movements, providing evidence of increases in views of financially related *Wikipedia* pages before stock market falls^25^. Evidence has also been provided that Google Trends data can be used to measure the risk of investment in a stock^26^.

Traders may however not only receive information through explicit attempts to search for information online, but by passively or actively receiving news broadcast by large financial news outlets. Equally, the actions of traders may lead to events which are described by the financial news. In this study, we seek to quantify the relationship between movements in financial news and movements in financial markets by exploiting a corpus of six years of financial news.

## Results

To examine the relationship between financial news and market behavior, we analyze a corpus of daily issues of the *Financial Times* from 2^nd^ January 2007 to 31^st^ December 2012. Details of how the corpus was retrieved and preprocessed are provided in the Supplementary Information.

**Figure 1:**
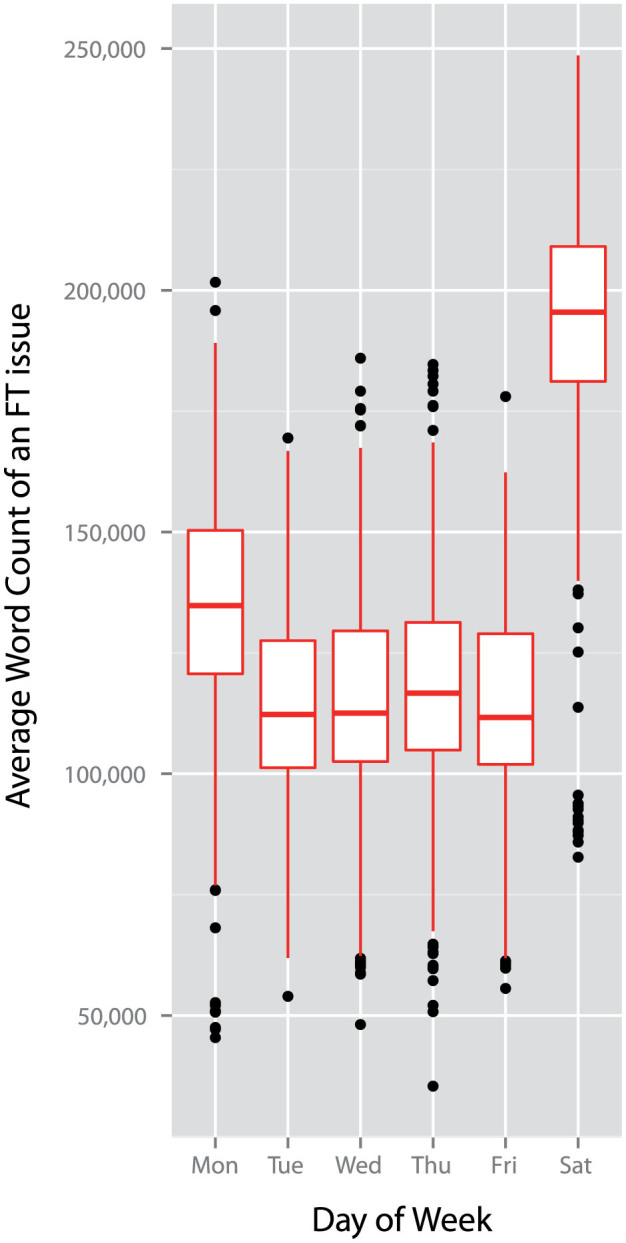
Daily variation in the total number of words occurring in each issue of the Financial Times. Daily variation in the total number of words in each issue of the Financial Times between 2^nd^ January 2007 and 31^st^ December 2012. We find significant differences in the length of the Financial Times on different days of the week (median of the number of total words for the given weekday: Monday, 134768.5; Tuesday, 112279; Wednesday, 112536; Thursday, 116690; Friday, 111663; Saturday, 195492; *χ*^2^ = 702.5324, *df* = 5, *p* < 0.001, Kruskal-Wallis rank sum test). Significantly longer issues are produced on Saturdays in comparison to the rest of the week (all *W*s > 128,000, all *p*s < 0.001, pairwise Wilcoxon rank sum tests with Bonferroni corrected *α* = 0.0033), and issues on Mondays are significantly longer than issues on Tuesday to Friday (all *W*s > 111,000, all *p*s < 0.001, pairwise Wilcoxon rank sum tests with Bonferroni corrected *α* = 0.0033). We find no evidence that the length of issues varies between Tuesday to Friday (all *W*s < 100,000, all *p*s > 0.01, pairwise Wilcoxon rank sum tests with Bonferroni corrected *α* = 0.0033).

The *Financial Times* is released each day from Monday to Saturday, at 5 am London time. An initial evaluation of the basic characteristics of the data, depicted in Figure 1, shows that there are significant differences in the length of the *Financial Times* on different days of the week (median of the number of total words for the given weekday: Monday, 134768.5; Tuesday, 112279; Wednesday, 112536; Thursday, 116690; Friday, 111663; Saturday, 195492; *χ*^2^ = 702.5324, *df* = 5, *p* < 0.001, Kruskal-Wallis rank sum test). We find longer issues on Saturdays in comparison to the rest of the week (all *W*s > 128,000, all *p*s < 0.001, pairwise Wilcoxon rank sum tests with Bonferroni corrected *α* = 0.0033), reflecting the publication of a special weekend edition of the *Financial Times*. Similarly, issues on Mondays, following the break on a Sunday, are significantly longer than issues on Tuesday to Friday (all *W*s > 111,000, all *p*s < 0.001, pairwise Wilcoxon rank sum tests with Bonferroni corrected *α* = 0.0033). We find no evidence that the length of issues varies between Tuesday to Friday (all *W*s < 100,000, all *p*s > 0.01, pairwise Wilcoxon rank sum tests with Bonferroni corrected *α* = 0.0033).

A total of 891,171 different words occur throughout the *Financial Times* corpus. We begin our investigation of the relationship between financial news and financial market movements by focusing on occurrences of the names of the 31 companies that were listed in the *Dow Jones Industrial Average* (DJIA) between 2^nd^ January 2008 and 31^st^ December 2012, a period for which we have transaction volume and price data for the DJIA components. At any one time, the DJIA consists of 30 companies. However, *Travelers* replaced *Citigroup* in the DJIA during the period of our analysis. In the calculations reported, we consider stock data and news data for both of these companies. Full details of the company names used in the corpus analysis are provided in the Supplementary Information (Table S1).

We investigate the relationship between interest in a company in the news and interest in a company in the stock markets. Stocks for companies listed in the DJIA are traded at the *New York Stock Exchange* (NYSE), open between 9:30 am and 4 pm New York time (for most of the year, 2:30 pm to 9 pm London time). We carry out this analysis and all following analyses for trading days only, excluding all weekends and bank holidays.

**Figure 2:**
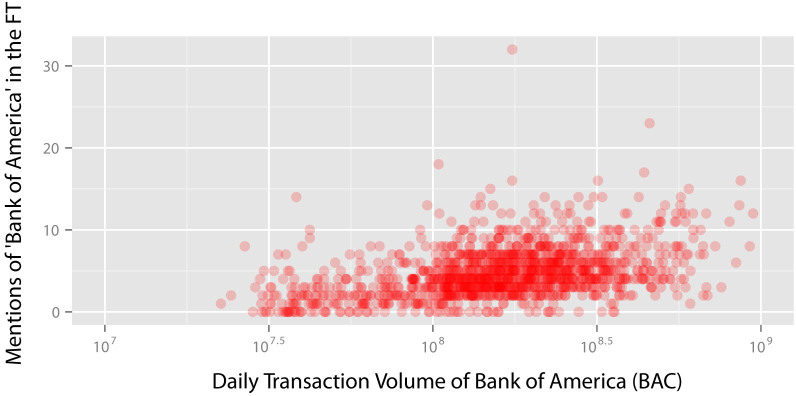
Daily number of mentions of “Bank of America” in the Financial Times and daily transaction volume for Bank of America (BAC) stocks. We depict the correlation between the daily number of mentions of “Bank of America” and the daily transaction volume for Bank of America (BAC) stocks. We find that the daily number of mentions of “Bank of America” is positively correlated with the daily transaction volume for Bank of America (BAC) stocks (*ρ* = 0.43, *p* < 0.001, Spearman's rank correlation).

In Figure 2, we take *Bank of America* as an example for this analysis, and plot the number of daily mentions of “Bank of America” against daily transaction volume for *Bank of America*. We find that a greater number of daily mentions of “Bank of America” corresponds to a greater daily transaction volume for *Bank of America* stocks (*ρ* = 0.43, *p* < 0.001, Spearman's rank correlation).

**Figure 3:**
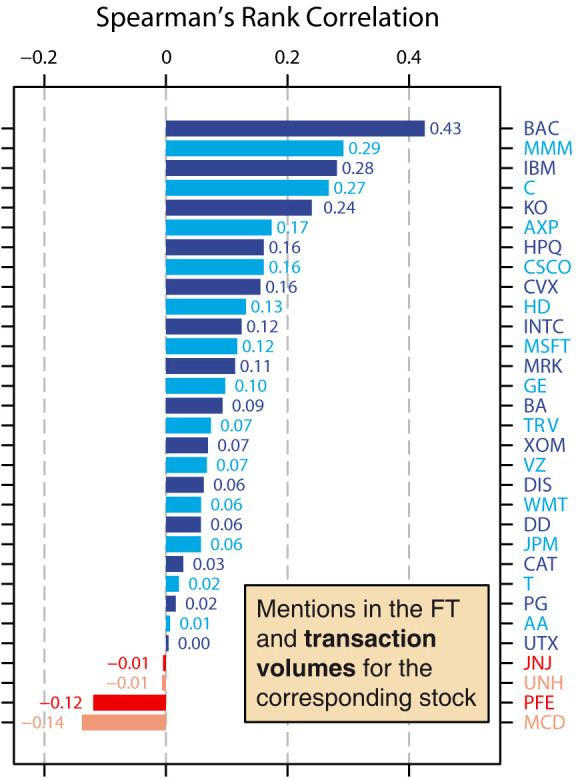
Correlations between daily mentions of a company's name and transaction volumes for the company's stock. For each of the 31 companies that were listed in the Dow Jones Industrial Average between 2^nd^ January 2008 and 31^st^ December 2012, we plot the Spearman's rank correlation between the daily number of mentions of a company's name and the transaction volume of the corresponding company's stocks. Companies are indicated using their ticker symbol, for which a full list can be found in the Supplementary Information (Table S1). We analyze the distribution of correlation coefficients and find that, overall, the correlation coefficients are significantly higher than zero (median correlation coefficient = 0.074; mean correlation coefficient = 0.100; *W* = 450, *p* < 0.001, Wilcoxon signed rank test). In other words, the daily number of mentions of a company's name is positively correlated with the daily transaction volume of a company's stocks.

We extend this analysis to all 31 *Dow Jones* companies from this period. For each company, we calculate the Spearman's rank correlation between the daily number of mentions of a company's name in the *Financial Times* and the transaction volume of the corresponding company's stocks (Figure 3). We analyze the distribution of Spearman's rank correlation coefficients for all 31 companies. Whilst the strongest correlation is found for *Bank of America*, we find that overall, the correlation coefficients are significantly higher than zero (median correlation coefficient = 0.074; mean correlation coefficient = 0.100, *W* = 450, *p* < 0.001, Wilcoxon signed rank test). A greater number of mentions of a company in the news therefore corresponds to a greater transaction volume of a company's stocks. This suggests greater interest in a company in the news is related to greater interest in a company in the stock markets.

We examine whether there is a similar link between the daily number of mentions of a company's name and the daily absolute return of the corresponding company's stocks. The absolute return indicates how much a stock price has changed, regardless of its direction. As a greater volume of trading is known to be correlated with greater movements in the price of a company's stock, it would be reasonable to expect the relationship between news and absolute return to be similar to the relationship we find between news and transaction volume.

**Figure 4:**
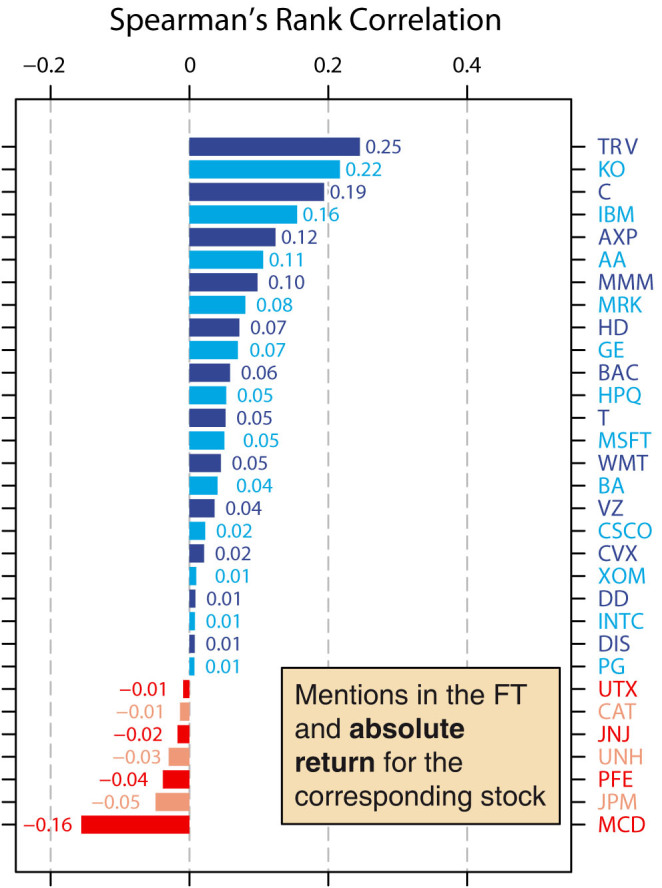
Correlations between daily mentions of a company's name and absolute return for the company's stock. We examine whether there is a link between the daily number of mentions of a company's name and the daily absolute return of the corresponding company's stocks. We calculate Spearman's rank correlation between the daily number of mentions and the daily absolute return, and again find that overall, the correlation coefficients are significantly higher than zero (median correlation coefficient = 0.040; mean correlation coefficient = 0.047; *W* = 408, *p* = 0.0017, Wilcoxon signed rank test). In other words, the daily number of mentions of a company's name is positively correlated with the daily absolute return of the company's stocks.

The daily return is defined as the natural logarithm of the ratio of the closing price of a given day to the closing price from the previous day. We compute the absolute daily return for each of the 31 companies by taking the absolute values of the daily returns, and calculate the Spearman's rank correlation between the daily number of mentions of a company and the company's daily absolute return (Figure 4). Again, we find that across all 31 companies, the correlation coefficients are significantly higher than zero (median correlation coefficient = 0.040; mean correlation coefficient = 0.047; *W* = 408, *p* = 0.0017, Wilcoxon signed rank test). Our results therefore suggest that greater interest in a company in the news is also related to greater movements in the company's stock price in the markets.

**Figure 5:**
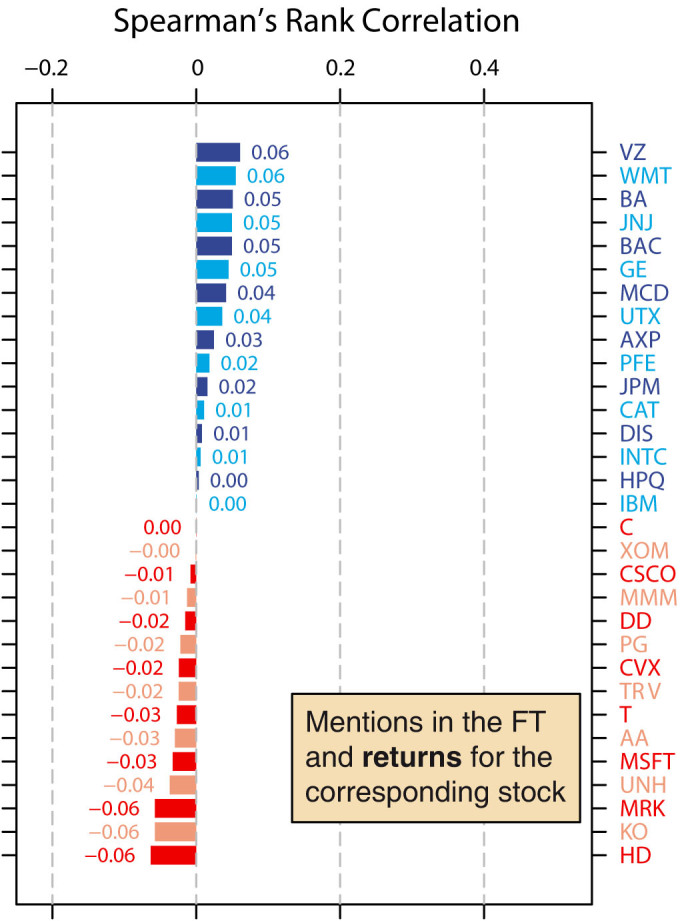
Correlations between daily mentions of a company's name and return for the company's stock. We investigate whether there is a relationship between the daily number of mentions of a company's name and the daily return of the corresponding company's stocks. Again, we calculate Spearman's rank correlation between the daily number of mentions and the daily return. Here, we find that the correlation coefficients are not significantly different to zero (median correlation coefficient = 0.000; mean correlation coefficient = 0.002; *W* = 262, *p* = 0.784, Wilcoxon signed rank test). In other words, the daily number of mentions of a company's name is not significantly correlated with the daily return of the company's stocks.

We investigate whether a relationship also exists between interest in a company in the news and movement in a company's stock price when direction of movement is taken into account. We calculate the Spearman's rank correlation between the daily number of mentions of a company and the daily return of a company's stocks (Figure 5), and find that here, the correlation coefficients are not significantly different to zero (median correlation coefficient = 0.000, mean correlation coefficient = 0.002, *W* = 262, *p* = 0.784, Wilcoxon signed rank test). In other words, our analysis so far provides no evidence that interest in a company in the news is correlated with company stock price movements when direction of movement is considered.

In summary, we find evidence for a relationship between interest in a company in the news on a given day, and both the volume of trading and size of price change for a company's stocks on the same day. We find no evidence for a relationship between interest in a company in the news on a given day and price change for a company's stocks on the same day when direction of this change is taken into account.

**Figure 6:**
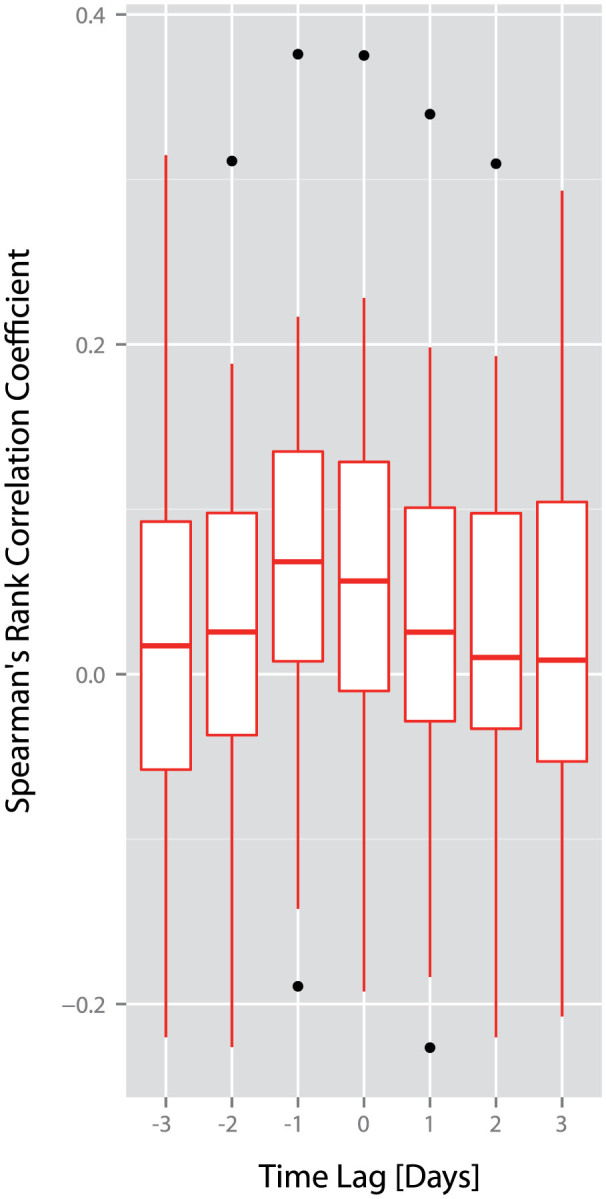
Lagged analysis of correlations between daily mentions of a company's name and transaction volumes for the company's stock. We investigate the correlation between daily mentions of a company's name and transaction volumes for the corresponding company's stock at different time lags. We calculate correlations between the daily number of mentions of a company's name and the daily transaction volume for a company from three days beforehand (indicated as −3 on the x-axis) to three days afterwards (indicated as 3 on the x-axis). We find that correlation coefficients for daily transaction volume one day before the news (−1) and on the same day as the news (0) are significantly greater than zero (lag −1: *W* = 373, *p* = 0.014; lag 0: *W* = 362, *p* = 0.026, Wilcoxon signed rank tests). In other words, a greater number of mentions of a company in the Financial Times is related to a greater transaction volume for a company's stocks on the same day and on the previous day. We find no significant relationship between the daily number of mentions of a company's name in the Financial Times and transaction volume at any other lag (lag −3: *W* = 270, *p* = 0.666; lag −2: *W* = 301, *p* = 0.299; lag 1: *W* = 317, *p* = 0.176; lag 2: *W* = 307, *p* = 0.248; lag 3: *W* = 298, *p* = 0.327; Wilcoxon signed rank tests).

However, whilst we are linking news released at 5 am London time on a given day with trading in a market much later in the day, between 9:30 am and 4 pm New York time, our current analyses do not allow us to draw strong conclusions about whether news influences the markets, or the markets influence the news. To gain some understanding of the directionality of this relationship, we extend this analysis by considering the relationship between mentions of a company in the news on a given day and transaction volume for a company on the three days beforehand, and the three days afterwards (Figure 6).

We find that correlation coefficients for daily transaction volume one day before the news (−1) and on the same day as the news (0) are significantly greater than zero (lag −1: *W* = 373, *p* = 0.014; lag 0: *W* = 362, *p* = 0.026, Wilcoxon signed rank tests). We find no significant relationship between the daily number of mentions of a company's name in the Financial Times and transaction volume at any other lag analyzed (lag −3: *W* = 270, *p* = 0.666; lag −2: *W* = 301, *p* = 0.299; lag 1: *W* = 317, *p* = 0.176; lag 2: *W* = 307, *p* = 0.248; lag 3: *W* = 298, *p* = 0.327; Wilcoxon signed rank tests). A greater transaction volume for a company's stocks on a given day is therefore related to a greater number of mentions of that company in the *Financial Times* on the following day. Equally, a greater number of mentions of a company in the *Financial Times* on a given day is related to a greater transaction volume for a company's stocks during trading later that day. Whilst more detailed analysis is required to draw strong conclusions about the direction of this relationship, these results are consistent with the hypothesis that movements in the news and movements in the markets may exert a mutual influence upon each other.

## Discussion

We use six years of daily print issues of the *Financial Times* to quantify the relationship between decisions taken in financial markets and developments in financial news. We analyze mentions of the companies that form the *Dow Jones Industrial Average*, and find that a greater number of mentions of a company in the news on a given morning corresponds to a greater volume of trading for that company during a given day, as well as a greater change in price for a company's stocks. Our analyses also uncover a link between the volume of trading for a company and the number of mentions of company in the news on the next day. Our current analysis provides no evidence of a relationship between the number of mentions of a company in the morning's news and the change in price for a company's stocks when direction of price movement is taken into account.

The results we present here are consistent with the hypothesis that movements in the news and movements in the markets may exert a mutual influence upon each other. Future analyses building on this work will seek to provide further insight into the direction and causality of the relationship between financial news and market movements.

## Methods

### Data retrieval and preprocessing

We analyze a corpus of daily issues of the *Financial Times* from 2^nd^ January 2007 to 31^st^ December 2012. We retrieved this data in PDF form from the website http://www.ft.com/. Issues of the *Financial Times* are released in PDF form online every day from Monday to Saturday at 5 am London time. All issues of the *Financial Times* were retrieved for this period, with the exception of five dates where technical problems prevented download. These dates were 22^nd^ February 2007, 8^th^ March 2007, 12^th^ May 2007, 28^th^ January 2009 and 8^th^ November 2012. In total, 1821 issues of the *Financial Times* are included in the analysis.

We preprocess the news data by converting the PDFs to text format, eliminating special characters such as ‘?', ‘−', ‘/' and converting all remaining words to lower case. All occurrences of digits without any letters or other symbols such as “$” in the same word are also removed. Words are not stemmed for this analysis, and stop words such as “the” and “and” are left in the corpus. We find 891,171 unique words in the corpus.

### Company names used for news corpus analysis

We compile a list of common names used for the 31 companies listed in the *Dow Jones Industrial Average* (DJIA) between 2^nd^ January 2008 and 31^st^ December 2012. At any one time, the *Dow Jones Industrial Average* (DJIA) is derived from the stock prices of 30 companies. On 8^th^ June 2009, during the period of our study, *Travelers* replaced *Citigroup* in the DJIA. In our analyses, we consider stock data and news data for both of these companies throughout the whole period.

To maximize the amount of news data available for our analysis, we determine commonly used forms of the names of the companies in the DJIA. We retrieved the names used to describe the companies on the *Wikipedia* page http://en.wikipedia.org/wiki/Dow_Jones_Industrial_Average on 21st May 2013. Where symbols such as “−” occur in these short names, we delete the symbol, and replace it with a space, if we find that this increases the number of hits for the name in the *Financial Times* corpus. The final list of short names used is given in Table S1 in the Supplementary Information.

### Statistical analyses of basic characteristics of the data

Our analyses focus on four types of time series for each of the 31 companies listed in the DJIA during the period of our study: the daily number of mentions of a company's name in the *Financial Times*, the daily transaction volume of a company's stock, the daily absolute return of a company's stock, and the daily return of a company's stock. Before running correlational analyses, we check for stationarity and normality of each of these 124 time series.

To check for stationarity, we first run an Augmented Dickey-Fuller test on each of these company name mention, daily transaction volume, daily absolute return and daily return time series. With the exception of the time series of mentions of *Coca-Cola* in the *Financial Times*, we reject the null hypothesis of a unit root for all time series, providing support for the assumption of stationarity of these time series (company names mentions: *Coca-Cola* *Dickey-Fuller* = −3.137, *p* = 0.099; all other *Dickey-Fuller* < −3.478, all other *p*s < 0.05; daily transaction volume: all *Dickey-Fuller* < −3.763, all *p*s < 0.05; daily absolute return: all *Dickey-Fuller* < −5.046, all *p*s < 0.01; daily return: all *Dickey-Fuller* < −9.371, all *p*s < 0.01). We verify the results of the Augmented Dickey-Fuller test with an alternative test for the presence of a unit root, the Phillips-Perron test. Here, we reject the null hypothesis of a unit root for all company name, transaction volume, absolute return and return time series, with no exceptions, again providing support for the assumption of stationarity of these time series (company names mentions: all *Dickey-Fuller Z*(*α*) < −1016.124, all *p*s < 0.01; daily transaction volume: all *Dickey-Fuller Z*(*α*) < −176.305, all *p*s < 0.01; daily absolute return: all *Dickey-Fuller* *Z*(*α*) < −1096.038, all *p*s < 0.01; daily return: all *Dickey-Fuller* *Z*(*α*) < −1118.684, all *p*s < 0.01).

To check for normality, we run a Shapiro-Wilk test on each of our company name mention, daily transaction volume, daily absolute return and daily return time series. We find that none of our 124 time series have a Gaussian distribution (company names mentions: all *W* < 0.945, all *p*s < 0.01; daily transaction volume: all *W* < 0.909, all *p*s < 0.01; daily absolute return: all *W* < 0.811, all *p*s < 0.01; daily return: all *W* < 0.962, all *p*s < 0.01). Throughout the study, we therefore test for the existence of relationships between datasets by calculating Spearman's rank correlation coefficient, a non-parametric measure which makes no assumption about the normality of the underlying data.

## Author Contributions

M.A., H.S.M. and T.P. performed analyses, discussed the results, and contributed to the text of the manuscript.

## Supplementary Material

Supplementary Information Supplementary Information
