# Supplementary material for: Quantifying the Relationship Between Financial News and the Stock Market
Source: Sci Rep. 2013 Dec 20;3:3578. doi: 10.1038/srep03578 (PMC3868958; doi:10.1038/srep03578)
Supplement: Supplementary Information [file srep03578-s1.pdf]

TITLE:

Supplementary Information: Quantifying the Relationship Between Financial News and the Stock Market

AUTHORS AND AFFILIATIONS:

Merve Alanyali[1*], Helen Susannah Moat[2] and Tobias Preis[2]

[1] Centre for Complexity Science, University of Warwick,
Coventry, CV4 7AL, UK
[2] Warwick Business School, University of Warwick,
Coventry, CV4 7AL, UK

* To whom correspondence should be addressed; E-mail: M.Alanyali@warwick.ac.uk

Table S1. Ticker symbols of the companies that form the DJIA and their common forms as used in news corpus analysis.

**Company Name**	**Ticker Symbol**	**Common Form**
Alcoa	AA	alcoa
American Express	AXP	american express
Boeing	BA	boeing
Bank of America	BAC	bank of america
Caterpillar	CAT	caterpillar
Cisco Systems	CSCO	cisco systems
Chevron Corporation	CVX	chevron
DuPont	DD	dupont
Walt Disney	DIS	walt disney
General Electric	GE	general electric
The Home Depot	HD	home depot
Hewlett-Packard	HPQ	hewlettpackard
IBM	IBM	ibm
Intel	INTC	intel
Johnson & Johnson	JNJ	johnson johnson
JPMorgan Chase	JPM	jpmorgan chase
Coca-Cola	KO	coca cola
McDonald’s	MCD	mcdonalds
3M	MMM	3m
Merck	MRK	merck
Microsoft	MSFT	microsoft
Pfizer	PFE	pfizer
Procter & Gamble	PG	procter gamble
AT&T	T	att
Travelers	TRV	travelers
United Health Group	UNH	unitedhealth group
United Technologies Corporation	UTX	united technologies
Verizon	VZ	verizon communications
Wal-Mart	WMT	walmart stores
ExxonMobil	XOM	exxon
Citigroup	C	citigroup
